# Gender and Obesity Specific MicroRNA Expression in Adipose Tissue from Lean and Obese Pigs

**DOI:** 10.1371/journal.pone.0131650

**Published:** 2015-07-29

**Authors:** Caroline M. Junker Mentzel, Christian Anthon, Mette J. Jacobsen, Peter Karlskov-Mortensen, Camilla S. Bruun, Claus B. Jørgensen, Jan Gorodkin, Susanna Cirera, Merete Fredholm

**Affiliations:** 1 Animal Genetics, Department of Veterinary Clinical and Animal Science, Faculty of Health Sciences, University of Copenhagen, Frederiksberg, Denmark; 2 Center for non-coding RNA in Technology and Health, Computational Biology and Bioinformatics, Department of Veterinary Clinical and Animal Science, Faculty of Health Sciences, University of Copenhagen, Frederiksberg, Denmark; Mayo Clinic Arizona, UNITED STATES

## Abstract

Obesity is a complex condition that increases the risk of life threatening diseases such as cardiovascular disease and diabetes. Studying the gene regulation of obesity is important for understanding the molecular mechanisms behind the obesity derived diseases and may lead to better intervention and treatment plans. MicroRNAs (miRNAs) are short non-coding RNAs regulating target mRNA by binding to their 3’UTR. They are involved in numerous biological processes and diseases, including obesity. In this study we use a mixed breed pig model designed for obesity studies to investigate differentially expressed miRNAs in subcutaneous adipose tissue by RNA sequencing (RNAseq). Both male and female pigs are included to explore gender differences. The RNAseq study shows that the most highly expressed miRNAs are in accordance with comparable studies in pigs and humans. A total of six miRNAs are differentially expressed in subcutaneous adipose tissue between the lean and obese group of pigs, and in addition gender specific significant differential expression is observed for a number of miRNAs. The differentially expressed miRNAs have been verified using qPCR. The results of these studies in general confirm the trends found by RNAseq. Mir-9 and mir-124a are significantly differentially expressed with large fold changes in subcutaneous adipose tissue between lean and obese pigs. Mir-9 is more highly expressed in the obese pigs with a fold change of 10 and a p-value < 0.001. Mir-124a is more highly expressed in the obese pigs with a fold change of 114 and a p-value < 0.001. In addition, mir-124a is significantly higher expressed in abdominal adipose tissue in male pigs with a fold change of 119 and a p-value < 0.05. Both miRNAs are also significantly higher expressed in the liver of obese male pigs where mir-124a has a fold change of 12 and mir-9 has a fold change of 1.6, both with p-values < 0.05.

## Introduction

Obesity is a risk factor for various complications and diseases such as hypertension, cardiovascular disease, dyslipidemia, inflammation, insulin resistance and type II diabetes. The obese state, characterized by excess fat accumulation, is in itself a burden to the affected subject with minor complications such as back pain, respiratory symptoms and difficulties in physical activity, but the secondary complications such as type 2 diabetes and coronary heart disease are expensive in terms of health-care costs as well as being life threatening [1].

The excess fat, in form of lipids, is stored in specialized cells (adipocytes) in the adipose tissue. Apart from storing lipids for later use as an energy source, adipose tissue functions as an endocrine organ secreting cytokines and interleukins involved in energy metabolism and inflammation. The endocrine activity derives from the adipocytes, but also from immune cells present in the adipose tissue. In obese subjects, macrophages are recruited to, and accumulate in the adipose tissue where they contribute to the inflammation seen in these subjects [2].

Excess lipids can be stored in different adipose depots, which have different functions. Abdominal or visceral adipose tissue (including retroperitoneal, omental and mesenteric fat) has more endocrine activity than subcutaneous adipose tissue [3]. An example is IL-6, an inflammatory mediator, which is secreted from both omental and subcutaneous adipose tissues in culture with 3-fold higher levels in the omental samples compared to the subcutaneous samples [4].

In humans, large amounts of fat in the waist area (central or abdominal obesity) is a greater risk predictor of obesity-derived complications than fat stored on the hip, thighs and buttocks (gluteo-femoral obesity) [5,6]. A simple measure of the waist circumference alone can indicate if there is a high risk of abdominal obesity related complications [7]. Gender greatly influences where fat is stored, whereas men store fat in the abdomen as visceral fat, women tend to store fat subcutaneously [8]. Men also have higher amounts of the deep subcutaneous adipose tissue, which is considered to be more metabolically active than the superficial subcutaneous adipose tissue, which is more thermo regulative. In contrast, women have equal amounts of deep subcutaneous adipose tissue and superficial adipose tissue [9].

Generally, subcutaneous adipose tissue is considered to be a healthier adipose tissue depot than visceral adipose tissue, but several studies have shown that the unhealthy state of the visceral adipose tissue is reflected in the subcutaneous adipose tissue, which in both pigs and humans have distinct layers of different metabolic activity [9–11]. Other studies also link the amount of subcutaneous tissue to insulin resistance regardless of the amount of visceral fat [12] and link dyslipidemia to both excess subcutaneous fat and increased liver fat [13].

Traditionally mouse biomedical models such as the knock out ob/ob mouse and diet induced obese mice have been used for obesity research, but results obtained in a rodent model are not always directly comparable to humans [14,15]. The pig is a more suitable model for human obesity due to its similarities in gastro-intestinal tract, organ-size, genetics, dietary habits and metabolism [15,16].

Previous sequencing studies of adipose tissue miRNA expression in pigs have been limited to comparison between a “lean” phenotype pig breed such as Landrace or Yorkshire, and an “obese” phenotype pig breed such as the Meichan or Lantang, or merely a comparison between adipose depots in a single pig [17–20]. In this study a well-characterized pig model designed for obesity studies has been used [21]. Briefly, the model is the F2 generation of a cross between lean production pigs, and Göttingen minipigs, which are prone to obesity. The outcome is a diverse population of lean, intermediate and obese pigs. The animals included in the present study have been selected to represent the most divergent phenotypes with respect to obesity traits.

MicroRNAs (miRNAs) are small (~21 nucleotides long) regulatory non-coding RNAs that bind to the 3’ UTR of the target mRNAs and suppress and/or degrade the mRNA thus inhibiting translation. MiRNAs originate from hairpin precursors (~70 nucleotides) which are processed into single stranded oligonucleotides which, when bound to a protein complex can base pair with its target mRNA by its ~7 nt long seed region. Each miRNA can regulate multiple target mRNAs and mRNAs may have multiple miRNA target sites [22,23].

It is estimated that more than 60% of all mammalian mRNA have conserved miRNA target sites in their 3’ UTR making protein suppression by miRNAs an important regulatory function in the cell [24].

MicroRNAs are involved in many obesity derived diseases including cardiovascular diseases, diabetes, metabolic syndrome and inflammation. Their expression has been found to correlate with different obesity relevant phenotypes such as body mass index (BMI), adipocyte size and metabolic parameters, implying that miRNAs play an important regulatory role in obesity [25–28].

In this study we performed next generation sequencing (NGS) of microRNA isolated from subcutaneous adipose tissue of lean and obese male and female pigs. To verify the sequencing results, qPCR was performed on the same samples. To increase functional information of the differentially expressed microRNAs qPCR was also performed on samples from abdominal adipose tissue, skeletal muscle and liver from the same animals.

## Methods

### Animal material

Fourteen pigs from the UNIK obesity resource population [21] were selected among the low and high extreme animals based on the physical obesity traits: BMI, abdominal circumference and amount of retroperitoneal fat measured at slaughter. Briefly, the population is the F2 generation of an intercross of Göttingen minipigs, which are naturally obesity prone, and Duroc and Yorkshire production pigs, which have been selected for leanness for decades. The F2 generation is phenotypically diverse showing various degrees of obesity when fed regular pig chow *ad libitum*. In this study F2 pigs originating from the intercross between Göttingen minipigs and Duroc were used. Of the 14 pigs, 7 had a lean phenotype (4 males + 3 females) and 7 had an obese phenotype (4 males + 3 females).

In Table 1 some of the key anthropometric and metabolic measures characterizing the phenotype of the selected pigs are provided. Details on the conformation traits are described in [21]. Briefly, retroperitoneal fat was bluntly excised at slaughter and the weight of it was corrected for body length; Body Mass Index (BMI) was calculated as weight (kg)/(length (cm))^2^; Body Adiposity Index (BAI) was calculated as abdominal circumference divided by length^1.5^; the abdominal circumference (ABC) was measured around the largest circumference of the body. With respect to the metabolic traits total cholesterol (ct) and triglycerides were measured in plasma collected at slaughter using commercial kits from Roche Diagnostics (Roche Diagnostics, USA) and ThermoElectron (Thermo Fisher Scientific Inc. France) respectively. ASAT and ALAT were measured on serum collected at slaughter using Advia 1800 Chemistry System (Siemens, Denmark). Fasting glucose was measured after 24 hours of fasting with a Freestyle Mini glucometer (Hermedico, Brøndby, Denmark) using a drop of blood from the ear vein.

**Table 1:** Mean values of the phenotypic measures of the lean and obese pigs calculated on all pigs, and the male and female pigs respectively.

	All			Males			Females		
	Lean	Obese	p-val	Lean	Obese	p-val	Lean	Obese	p-val
**Number**	7	7		4	4		3	3	
**Age (months)**	8.77 ± 3	8.59 ± 1.4	0.890	10.77 ± 2.4	8.15 ± 1.7	0.120	6.1 ± 0.5	9.2 ± 0.5	0.002
**RP_Fat (kg/cm)**	1.67 ± 1	4.24 ± 1.36	<0.005	0.95 ± 0.65	3.41 ± 0.93	0.005	2.64 ± 0.29	5.36 ± 0.97	0.010
**BMI (kg/m^2^)**	111 ± 7.6	152 ± 10.2	<0.005	106 ± 7.9	151 ± 11.9	<0.005	116 ± 4.6	154 ± 12	0.007
**BAI (cm/cm^1.5^)**	0.14 ± 0.01	0.16 ± 0.019	0.026	0.13 ± 0.01	0.16 ± 0.02	0.063	0.15 ± 0.02	0.16 ± 0.01	0.268
**ABC (cm)**	112 ± 10.6	134 ± 11	<0.005	105 ± 7	131 ± 9	<0.005	120 ± 8	138 ± 14	0.127
**Weight (kg)**	85 ± 14	120 ± 26	0.008	82 ± 10	117 ± 26	0.046	88.5 ± 19	124 ± 31	0.166
**Ct (mM)**	2.34 ± 0.65	2.64 ± 0.53	0.370	2.10 ± 0.6	2.2 ± 0.4	0.400	2.66 ± 0.7	2.93 ± 0.6	0.650
**TG (mM)**	0.49 ± 0.2	0.51 ± 0.32	0.877	0.38 ± 0.1	0.40 ± 0.07	0.754	0.64 ± 0.21	0.67 ± 0.49	0.930
**Fasting_glu (mM)**	3.22 ± 0.76	3.48 ± 0.82	0.570	3.15 ± 1	3.36 ± 0.8	0.780	3.33 ± 0.15	3.6 ± 0.98	0.670
**ASAT**	51 ± 14	83 ± 36	0.050	54 ± 17.5	96 ± 35	0.074	47 ± 7.4	65 ± 36	0.445
**ALAT**	43 ± 7	57 ± 38	0.315	40 ± 6.5	43 ± 7.3	0.504	46 ± 6,6	77 ± 57	0.403
**ASAT/ALAT**	1.21 ± 0.21	1.66 ± 0.73	0.012	1.34 ± 0.2	2.2 ± 0.55	0.026	1.04 ± 0.14	0.95 ± 0.26	0.63

RP_Fat: weight of the retroperitoneal fat corrected for length of the pig; BMI: Body Mass Index calculated as weight (kg)/(length (m))^2^; BAI: Body Adiposity Index calculated as abdominal circumference divided by length^1.5^; ABC: the abdominal circumference; TG: triglycerides; Ct: total cholesterol; Fasting_glu is the glucose level measured after 24H of fasting. ASAT: aspartate amino transferase (U/L). ALAT: alanine amino transferase (U/L). ASAT/ALAT: ratio of ASAT and ALAT. All values are displayed as mean ± SD.

The project was approved by the Danish Animal Experimentation Board, Animal care and maintenance have been conducted according to the Danish “Animal Maintenance Act” (Act 432 dated 09/06/2004). The animals were housed at a regular pig farm, and slaughtered at a commercial slaughterhouse by stunning and bleeding under veterinary supervision as required by the Danish authorities. Tissue and blood samples were collected at slaughter.

### RNA purification

Subcutaneous adipose tissue, abdominal adipose tissue, muscle and liver (among other tissues) were collected from all animals at slaughter, snap frozen in liquid nitrogen and stored at -80°C until RNA extraction.

RNA from adipose tissues was extracted using an in house protocol combining Tri Reagent and the Qiagen miRNeasy kit [29]. Briefly, 100 mg adipose tissue were homogenized in Tri Reagent on a gentleMACS Octo Dissociator (Miltenyi Biotec, Germany) following manufacturer’s instructions. The homogenate was centrifuged and visible fat and cell debris were removed. Chloroform was added and the sample was centrifuged to separate the nucleic acid and protein phases. The RNA phase was mixed with ethanol and transferred to a Qiagen miRNeasy spin column and RNA was purified according to the miRNeasy protocol. Finally RNA was eluted with RNase-free water.

RNA from muscle and liver was extracted using the Tri Reagent protocol (MRCgene, Molecular Research Center, Inc, US).

RNA concentration and purity was measured using a Nanodrop ND-1000 spectrophotometer (NanoDrop technologies, Wilmington, USA). RNA integrity was assessed in an Experion machine (Biorad) and samples with RQI ≥ 7 were accepted for further analysis (Subcutaneous adipose tissue: RQI: 7.99 ± 0.47, A_260/280_: 2.07 ± 0.05; Abdominal adipose tissue: RQI: 8.44 ± 0.5 A_260/280_: 1.96 ± 0.16; Liver: RQI: 9.73 ± 0.22 A_260/280_: 1.96 ± 0.05; Muscle: RQI: 9.05 ± 0.44 A_260/280_: 1.87 ± 0.05. All values represent the mean ± SD).

### Illumina sequencing

Small RNA libraries were prepared from 1 μg total RNA from subcutaneous adipose tissue by the standard TruSeq small RNA sample preparation protocol (Part # 15004197 Rev. E) following manufacturer’s instructions. Briefly, RNA was ligated to a 3’ adaptor followed by ligation to a 5’ adaptor. RNA was reversely transcribed and amplified using TruSeq indexes to barcode the samples. The libraries were size-selected by 3% MetaPhor agarose gel (Cambrex Bio Science, Rockland) for the small RNA fraction including miRNAs and sequenced on two lanes on an Illumina Genome Analyzer IIx.

### Sequence data analysis

Sequencing of miRNAs from 14 samples produced 52,883,376 reads. Fastq files were trimmed for adaptors and for bases with a quality score below 20 by the trimming program Cutadapt [30]. Sequences were filtered for length using a custom Perl script and sequences with a length of 18–25 nucleotides (representing miRNAs) were used for further analysis. This left 44,606,110 reads.

The miRDeep2 package [31] was used for mapping the reads to the genome and to quantify all known porcine miRNAs (Mirbase v 20 [32]) in the dataset. 37,529,528 reads mapped to the porcine genome sequence [16]. The mirDeep2 output with miRNA counts can be found in S1 Dataset.

The DESeq2 package in the statistics software R (Version 3.0.1) was used for differential expression analysis with standard parameters [33]. The result of the DESeq2 analysis for all animals, males and females can be found in S2–S4 Datasets.

### cDNA synthesis

Total RNA was used for cDNA synthesis as previously described [34,35]. For subcutaneous adipose tissue the same RNA samples used for sequencing were used for qPCR. Briefly, for miRNA analysis, two cDNA replicates per sample were made from 100 ng of RNA in a final volume of 10 μl including 1 μl of 10x poly(A) polymerase buffer, 0.1 mM of ATP, 1 μM of RT-primer, 0.1 mM of each deoxynucleotide (dATP, dCTP, dGTP and dTTP), 100 units of MuLV reverse transcriptase (New England Biolabs, USA) and 1 unit of poly(A) polymerase (New England Biolabs, USA) were incubated at 42°C for 1 hour followed by enzyme inactivation at 95°C for 5 minutes. The sequence of the RT-primer was 5’-CAGGTCCAGTTTT TTTTTTTTTTTVN, where V is A, C and G and N is A, C, G and T. The primer was purchased from TAG Copenhagen (Denmark).

For mRNA analysis two cDNA replicates were made from 100 ng RNA for each sample in a final volume of 10 μl including 0.25 μg 3:1 mixture of random hexamers/OligodT, 2 μl Improm-II buffer, 2 mM dNTP mix, 10 units RNasin Ribonuclease inhibitor, 1.25 mM MgCl_2_ and 0.5 μl Improm-II reverse transcriptase (Promega), which were incubated for 42°C for 1 hour and 15 min at 70°C following manufacturer’s instructions.

### Primer design

All miRNA qPCR primers were designed according to the miRprimer design rules as previously described [34–36]. Briefly, Tm of both forward and reverse primer was optimized to 59°C by adjusting the tail length of the primers. Melting temperature (Tm) was calculated according to the nearest-neighbor model [37]. The primers were designed using the publicly available software miRprimer [36]. Sequences of primers and templates are given in Table 2.

**Table 2:** Primer sequences for qPCR detection of miRNA.

miRNA	mirbase ID	forward primer	reverse primer
**ssc-let-7a**	MIMAT0013865	GCAGTGAGGTAGTAGGTTGT	GGTCCAGTTTTTTTTTTTTTTTAACTATAC
**ssc-mir-1**	MIMAT0010187	CGCAGTGGAATGTAAAGAAGT	GGTCCAGTTTTTTTTTTTTTTTACATAC
**ssc-mir-9-1**	MIMAT0002168	GCAGTCTTTGGTTATCTAGCTGT	GGTCCAGTTTTTTTTTTTTTTTCATAC
**ssc-mir-10b**	MIMAT0013885	CAGTACCCTGTAGAACCGA	GGTCCAGTTTTTTTTTTTTTTTACAAATTC
**ssc-miR-16**	MIMAT0007754	GCTGTAGCAGCACGTA	CAGTTTTTTTTTTTTTTTCGCCAAT
**ssc-miR-17-5p**	MIMAT0007755	CAAAGTGCTTACAGTGCAG	GGTCCAGTTTTTTTTTTTTTTTCTAC
**ssc-miR-20a**	MIMAT0002129	ACAGTAAAGTGCTTATAGTGCA	GTCCAGTTTTTTTTTTTTTTTCTACCT
**ssc-mir-24-3p**	MIMAT0002134	GGCTCAGTTCAGCAGGA	GGTCCAGTTTTTTTTTTTTTTTCTG
**ssc-miR-26a**	MIMAT0002135	GCAGTTCAAGTAATCCAGGATAG	GTCCAGTTTTTTTTTTTTTTTAGCCT
**ssc-miR-27b-3p**	MIMAT0013890	CAGTTCACAGTGGCTAAGTTC	TCCAGTTTTTTTTTTTTTTTGCAGA
**ssc-miR-99a**	MIMAT0013896	CAGAACCCGTAGATCCGA	GGTCCAGTTTTTTTTTTTTTTTCAC
**ssc-miR-99b**	MIMAT0006018	CCCGTAGAACCGACCT	TCCAGTTTTTTTTTTTTTTTCGCA
**ssc-miR-100**	MIMAT0013911	GAACCCGTAGATCCGAAC	GGTCCAGTTTTTTTTTTTTTTTCAC
**ssc-miR-103**	MIMAT0002154	AGAGCAGCATTGTACAGG	GGTCCAGTTTTTTTTTTTTTTTCATAG
**ssc-mir-107**	MIMAT0002155	GCAGAGCAGCATTGTACAG	GGTCCAGTTTTTTTTTTTTTTTGATAG
**ssc-mir-124a**	MIMAT0002156	AGGCACGCGGTGA	CCAGTTTTTTTTTTTTTTTGGCATTC
**ssc-miR-133a-3p**	MIMAT0010186	TGGTCCCCTTCAACCAG	GGTCCAGTTTTTTTTTTTTTTTCAG
**ssc-miR-133b**	MIMAT0013869	TGGTCCCCTTCAACCAG	GTCCAGTTTTTTTTTTTTTTTATAGCTG
**ssc-mir-143-3p**	MIMAT0013879	CAGTGAGATGAAGCACTGT	TCCAGTTTTTTTTTTTTTTTGAGC
**ssc-miR-148a-3p**	MIMAT0002124	CAGTCAGTGCACTACAGAAC	GGTCCAGTTTTTTTTTTTTTTTACAAAG
**ssc-miR-191**	MIMAT0013876	AACGGAATCCCAAAAGCA	TCCAGTTTTTTTTTTTTTTTCAGC
**ssc-miR-199a-5p**	MIMAT0013874	CCCAGTGTTCAGACTACCTG	GTCCAGTTTTTTTTTTTTTTTGAACAG
**ssc-mir-215**	MIMAT0010192	CGCAGATGACCTATGAATTGAC	GGTCCAGTTTTTTTTTTTTTTTGTCT
**ssc-miR-221-3p**	MIMAT0007762	CAGAGCTACATTGTCTGCTG	TCCAGTTTTTTTTTTTTTTTAAACCCA
**TGFB1**		CGAGCCCTGGATACCAACT	GCAGAAATTGGCATGGTAG
**ITGB1**		GGTGAATGGGAACAATGAGG	GCAAGGCCAATGAGAACAAT
**NFKBIA**		GAGGATGAGCTGCCCTATGAC	CCATGGTCTTTTAGACACTTTCC
**NFKB1**		CTCGCACAAGGAGACATGAA	GGGTAGCCCAGTTTTTGTCA
**IL6R**		GTGCAGCTCAGTGACTCTGG	TCCACTCACAGCCCACATT
**ETS1**		CAGGAGATGGCTGGGAATTCA	CGTTTACCCGCCGTCTTGT
**ADIPOR2**		ATGGCCAGCCTCTACATCAC	GCCATGGAAGTGAACGAAAG
**SIRT1**		ACCAGAGCAGTTTCATAGAGCC	CAGGTGAGGCAAAGGTTCCC

MiRNA name, mirbase ID and forward and reverse primers for qPCR detection.

Quantitative PCR Messenger RNA primers were designed using PRIMER 3 software (http://bioinfo.ut.ee/primer3-0.4.0/). If possible, primers were designed over introns. Primer sequences are listed in Table 2. Primer sequences for NFKB1 and NFKB1a were as described in [38] and TGFB1 from [39]. Primers were purchased from Sigma (UK).

Quantitative PCR for miRNAs and mRNAs was performed on a MX3005P machine (Stratagene, USA). 1 μl of cDNA diluted 8 times, 5 μl of 2x QuantiFast SYBR Green PCR master mix (Qiagen, Germany), 250 nM of each primer (Table 2) were mixed in a final volume of 10 μl. Standard curves with 5-fold dilutions (made with a pool of equal amounts of cDNA from the 14 samples included in the study) were made for all primer sets to calculate qPCR efficiency. Cycling conditions were 95°C for 5 min followed by 40 cycles of 95°C for 10 sec and 60°C 30 sec. A melting curve analysis (60°C to 99°C) was performed after the thermal profile to ensure specificity of the assays.

### qPCR data analysis

Quantification was based on determination of the quantification cycle (Cq). Data was analyzed using Genex5 Pro (MultiD, Göteborg, Sweden). Cq values were corrected using the PCR efficiency, which was calculated from the log-linear portion of the standard curves [40]. Potential reference genes (let-7a, mir-17 and mir-26a for subcutaneous adipose tissue and mir-17 and mir-20 for abdominal adipose tissue, liver and skeletal muscle) were tested for stability using GeNorm [41] and NormFinder [42] algorithms and were all used as reference genes to normalize the expression of the individual samples in the qPCR experiment. GeNorm and NormFinder values for the reference genes and boxplots for the raw Ct expression of reference genes in obese and lean animals are available in S9 Dataset. Technical replicates were averaged and relative quantities where calculated by setting the lowest expressed sample in each primer assay to 1. Data was log_2_ transformed to achieve normality before using parametric methods (Student’s t-test). All graphs were made in Prism 6 (GraphPad Software, Inc. CA, USA) as column scatter plots where individual values within the groups are plotted together with indication of the mean and standard deviation. QPCR data is available in S5, S6, S8 and S9 Datasets.

### Target gene search

MiRWalk [43] and miRTarBase [44] databases were searched for experimentally verified miRNA targets. In addition MiRWalk was also searched for predicted targets using 8 different algorithms. DAVID tools [45,46] were used for mining the target genes for genes relevant for obesity-derived diseases, such as diabetes type II and heart disease. A list of verified target genes is included in S7 Dataset.

## Results

The pig population used in this study consists of 14 pigs (6 females and 8 males). The pigs were selected from the population of F2 pigs to form two groups (7 + 7 pigs, with 3 females and 4 males in each group) with a lean and an obese phenotype respectively. Anthropomorphic and metabolic measures of the selected pigs are presented in Table 1. The groups differ significantly in several of the physical parameters measured in the F2 population such as amount of abdominal (retroperitoneal) fat, Body Mass Index, Body Adiposity index and weight.

Sequencing of the small RNA fraction isolated from subcutaneous adipose tissue from the 14 samples produced 52,883,376 reads. After filtering the quality of the sequence, adaptors and read length 43,697,000 reads remained and of those 37,529,528 reads mapped to the porcine genome. The read counts for all the analyzed miRNAs can be seen in S1 Dataset.

**Fig 1:**
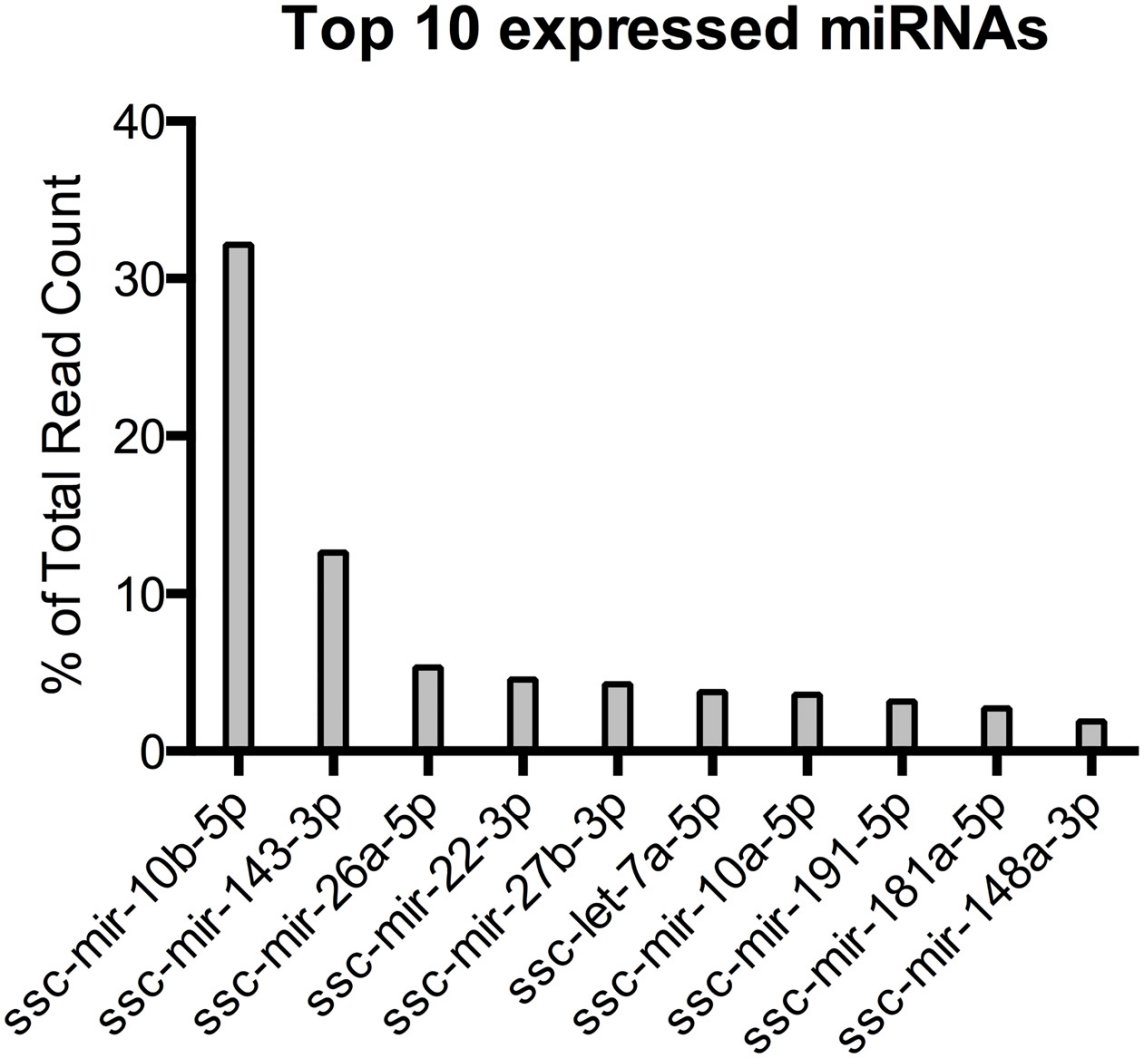
Top 10 expressed miRNAs in the sequencing study. The read count represents the total, un-normalized, read count for each miRNA from a pool of all the 14 animals in the study. % of Total Read Count is the percentage of the total reads of 37,529,528 that map to the porcine genome. The top 10 most expressed miRNAs account for more than 74% of the total read count.

The sequences were mapped to known porcine miRNAs by mirDeep2. The top 10 most abundant miRNAs found in the subcutaneous adipose tissue are illustrated by total read count in Fig 1. As seen in other miRNA sequencing studies [17,20,47] the highest expressed miRNAs accounts for a large percentage of the total read count. In the present study the top 10 most abundant miRNAs represent 74% of the read counts, and one miRNA (mir-10b) account for more than 32% of the reads. These highly expressed miRNAs are also found in other adipose tissue miRNA sequencing studies and may have important housekeeping functions in adipose tissue [17,20,47].

Subsequently, analysis for differentially expressed miRNAs between the obese and lean group was performed using DESeq2 in R. A total of 6 miRNAs were significantly differently expressed between the two groups with an adjusted p-value (adjusted for multiple testing using the Benjamini and Hochberg method in DESeq2) cutoff of 0.05 (Table 3).

**Table 3:** Top 10 differentially expressed genes for the combined dataset of lean versus obese pigs as analyzed by DESeq2.

miRNA	baseMean	log2FC	FC	p-value	p_adj_
**ssc-mir-9-1-5p**	299	3.20	9.19	5.57E-13	1.56E-11
**ssc-mir-124a-1-3p**	31.3	3.38	10.40	4.21E-11	1.06E-09
**ssc-mir-9-1-3p**	6.4	2.21	4.61	4.27E-06	9.36E-05
**ssc-mir-199a-1-5p**	6333	-1.16	-2.24	2.56E-05	0.0004
**ssc-mir-489-3p**	3.9	1.74	3.33	0.0009	0.013
**ssc-mir-34c-1-3p**	5	1.69	3.23	0.0019	0.027
**ssc-mir-4332-3p**	16	0.89	1.85	0.0052	0.069
**ssc-mir-148a-3p**	30708	-0.38	-1.30	0.0054	0.070
**ssc-mir-145-3p**	497	-0.56	-1.47	0.0062	0.077
**ssc-mir-153-3p**	2.6	1.41	2.66	0.0064	0.077

BaseMean: normalized mean read count for all animals; Log2FC: log2 fold change. FC: fold change; P_adj_: p-value adjusted for multiple testing (Benjamini and Hochberg as default by DESeq2). A positive fold change indicates that the miRNA is up regulated in obesity and a negative indicate down regulation.

The analysis was also performed on only lean versus obese male pigs (Table 4) and lean versus obese female pigs (Table 5). For the males we found 7 differentially expressed miRNAs, two of which (mir-9-1-3p and mir-199a-5p) overlap with the analysis of the combined dataset.

**Table 4:** Significantly differentially expressed genes in for lean versus obese male pigs as analyzed by DESeq2.

miRNA	baseMean	log2FC	FC	p-value	p_adj_
**ssc-mir-199b-5p**	3974	-0.87	-1.83	2.64E-08	6.22E-06
**ssc-mir-199a-5p**	7021	-1.28	-2.43	2.45E-05	1.15E-03
**ssc-mir-130a-3p**	530	-0.92	-1.89	3.89E-05	1.67E-03
**ssc-mir-181c-5p**	1323	-0.62	-1.54	3.27E-04	1.22E-02
**ssc-mir-9-1-3p**	5	1.94	3.83	3.89E-04	1.22E-02
**ssc-mir-205-5p**	21	-1.80	-3.47	6.10E-04	1.80E-02
**ssc-mir-455-3p**	211	0.81	1.76	8.43E-04	2.17E-02

BaseMean: normalized mean read count for all animals; Log2FC: log2 fold change. FC: fold change; P_adj_: p-value adjusted for multiple testing (Benjamini and Hochberg as default by DESeq2) Significance cut off is p_adj_ = 0.05. A positive fold change indicates that the miRNA is up regulated in obesity and a negative indicate down regulation.

**Table 5:** Significantly differentially expressed genes for lean versus obese female pigs as analyzed by DESeq2.

miRNA	baseMean	log2FC	FC	p-value	p_adj_
**ssc-mir-10b-5p**	588019	-1.14	-2.21	4.08E-30	1.99E-27
**ssc-mir-125a-5p**	11407	-0.85	-1.80	7.51E-28	1.83E-25
**ssc-mir-10a-5p**	72498	-1.42	-2.68	8.43E-15	1.37E-12
**ssc-mir-124a-3p**	32	3.05	8.30	1.19E-11	1.16E-09
**ssc-mir-340-5p**	194	0.90	1.86	6.52E-09	4.52E-07
**ssc-mir-342-3p**	118	-1.14	-2.20	7.41E-09	4.52E-07
**ssc-mir-125b-5p**	6582	-0.81	-1.76	1.06E-07	5.16E-06
**ssc-mir-574-3p**	584	-0.53	-1.44	3.08E-07	1.16E-05
**ssc-mir-148a-3p**	24953	-0.64	-1.56	3.75E-07	1.31E-05
**ssc-mir-151-5p**	8007	-0.57	-1.48	1.56E-06	5.09E-05
**ssc-mir-30a-5p**	18751	-0.49	-1.40	2.29E-06	6.99E-05
**ssc-mir-191-5p**	64358	-0.98	-1.97	2.82E-06	8.11E-05
**ssc-mir-100-5p**	10114	-1.45	-2.74	6.05E-06	1.64E-04
**ssc-mir-128-3p**	348	0.75	1.68	8.96E-06	2.06E-04
**ssc-mir-150-5p**	403	-0.74	-1.67	1.12E-05	2.27E-04
**ssc-mir-22-3p**	60767	0.34	1.27	5.11E-05	9.97E-04
**ssc-mir-2320-5p**	203	-0.90	-1.86	5.93E-05	1.11E-03
**ssc-mir-99a-5p**	5018	-1.03	-2.04	6.13E-05	1.11E-03
**ssc-mir-365-3p**	604	-0.68	-1.60	8.33E-05	1.40E-03
**ssc-mir-127-3p**	3944	-1.16	-2.24	1.51E-04	2.39E-03
**ssc-mir-151-3p**	8671	-0.59	-1.50	1.57E-04	2.39E-03
**ssc-mir-26a-5p**	65975	-0.30	-1.23	1.66E-04	2.46E-03
**ssc-mir-769-5p**	308	0.54	1.46	1.32E-03	1.85E-02
**ssc-mir-182-5p**	382	-1.43	-2.70	1.43E-03	1.93E-02
**ssc-mir-23a-3p**	2939	-0.39	-1.31	1.92E-03	2.29E-02
**ssc-mir-30c-5p**	2208	-0.38	-1.31	1.88E-03	2.29E-02
**ssc-mir-103-3p**	7213	0.67	1.60	3.24E-03	2.58E-02
**ssc-mir-214-3p**	652	-0.36	-1.28	3.11E-03	2.58E-02
**ssc-mir-218-5p**	141	0.82	1.77	3.38E-03	2.58E-02
**ssc-mir-361-5p**	300	-0.52	-1.44	3.80E-03	2.86E-02
**ssc-mir-450a-5p**	35	-0.94	-1.92	4.04E-03	2.99E-02
**ssc-mir-450c-5p**	57	-0.85	-1.81	4.35E-03	2.99E-02
**ssc-mir-7134-5p**	106	-0.58	-1.49	4.24E-03	2.99E-02
**ssc-mir-9-1-3p**	8	1.58	2.99	4.60E-03	2.99E-02
**ssc-mir-99b-5p**	10899	-1.06	-2.08	6.29E-03	4.04E-02

BaseMean: normalized mean read count for all animals; Log2FC: log2 fold change. FC: fold change; P_adj_: p-value adjusted for multiple testing (Benjamini and Hochberg as default by DESeq2) Significance cut off is p_adj_ = 0.05. A positive fold change indicates that the miRNA is up regulated in obesity and a negative indicate down regulation.

In the analysis of the female pigs 35 miRNAs were differentially expressed between the lean and obese animals (Table 5). Among these the expression profiles of mir-124a-3p and mir-9-3p overlapped with the results for the analysis of the combined dataset. All p-values for the DESeq2 analysis for the combined dataset as well as the male and female analysis can be found in S2–S4 Datasets.

**Fig 2:**
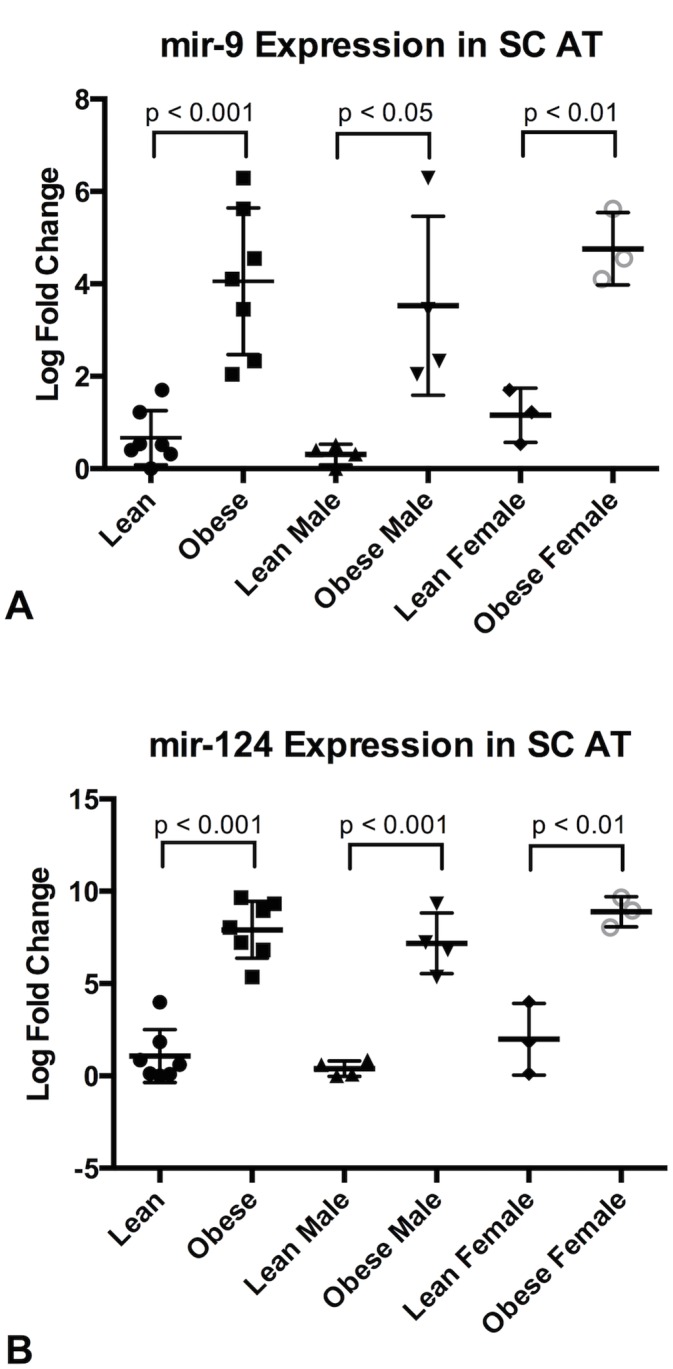
Expression of mir-9 and mir-124a in Subcutaneous Adipose Tissue. qPCR expression of mir-9 (A) and mir-124a (B) in Subcutaneous Adipose Tissue (SC AT). Column scatter plot of the groups Lean and Obese, Lean Male and Obese Male and Lean Female and Obese Female is shown for each of the three microRNAs. P-values calculated by Student’s t-test.

The most differentially expressed miRNAs found in the sequencing study together with a selection of potential obesity relevant miRNAs selected from the literature were verified by qPCR (Table 6). Generally the expression pattern followed what was seen in the NGS sequencing data, i.e. a miRNA up regulated in obese pigs in the RNAseq study was also up regulated when analyzed by qPCR. An exception from this general trend was mir-199-5p which was not significantly differentially expressed in the qPCR study. Also mir-489 and mir-34c could not be detected in all animals due to very low expression, and were therefore excluded from the qPCR analysis. Two of the 6 miRNAs found to be differentially expressed in the sequencing study, mir-9 and mir-124a, were significantly differentially expressed between the two groups (Table 6, Fig 2). Both the 3’ and 5’ mature miRNAs of mir-9 were significantly differentially expressed in the sequencing analysis, but only mir-9-5p was tested in qPCR due to low expression of mir-9-3p.

**Table 6:** qPCR results from lean and obese pigs in all pigs, and the male and female pigs respectively.

	All			Males			Females		
	FC	Log2FC	p-value	FC	Log2FC	p-value	FC	Log2FC	p-value
**Mir-124a**	114.43	6.84	1.82E-06	110.66	6.79	0.0002	119.66	6.90	0.005
**Mir-9**	10.43	3.38	1.90E-04	9.31	3.22	0.016	12.15	3.60	0.003
**Mir-103**	1.35	0.43	0.08	1.12	0.16	0.60	1.73	0.79	0.03
**Mir-99a**	-1.08	-0.11	0.45	-1.35	-0.43	0.03	1.24	0.31	0.04
**Mir-10b**	-1.11	-0.15	0.40	-1.31	-0.39	0.18	1.13	0.17	0.04

FC: fold change; Log2FC: log2 of fold change; p-value calculated by Student’s t-test. A positive fold change indicates that the miRNA is up regulated in obesity and a negative indicate down regulation.

When the qPCR results were analyzed for male pigs only, mir-99a was differentially expressed in addition to mir-124a and mir-9 (Table 6). In the female pigs mir-9, mir-124a, mir-103, mir-10b and mir-99a were differentially expressed (Table 6). Interestingly, in the male pigs mir-99a was down regulated in the obese subjects, while for the females mir-99a was up regulated in the obese subjects.

In Fig 2 the expression of mir-9 and mir-124a in lean and obese subcutaneous adipose tissue is illustrated in a Column scatter plot as the relative mean of log2 fold change were the lowest expressed sample of each miRNA assay is set to 1. Mir-9 and mir-124a are both significantly up regulated in obese animals with even higher fold changes than found in the sequencing study. Fold changes and p-values for qPCR in subcutaneous adipose tissue can be found in Table 6.

**Fig 3:**
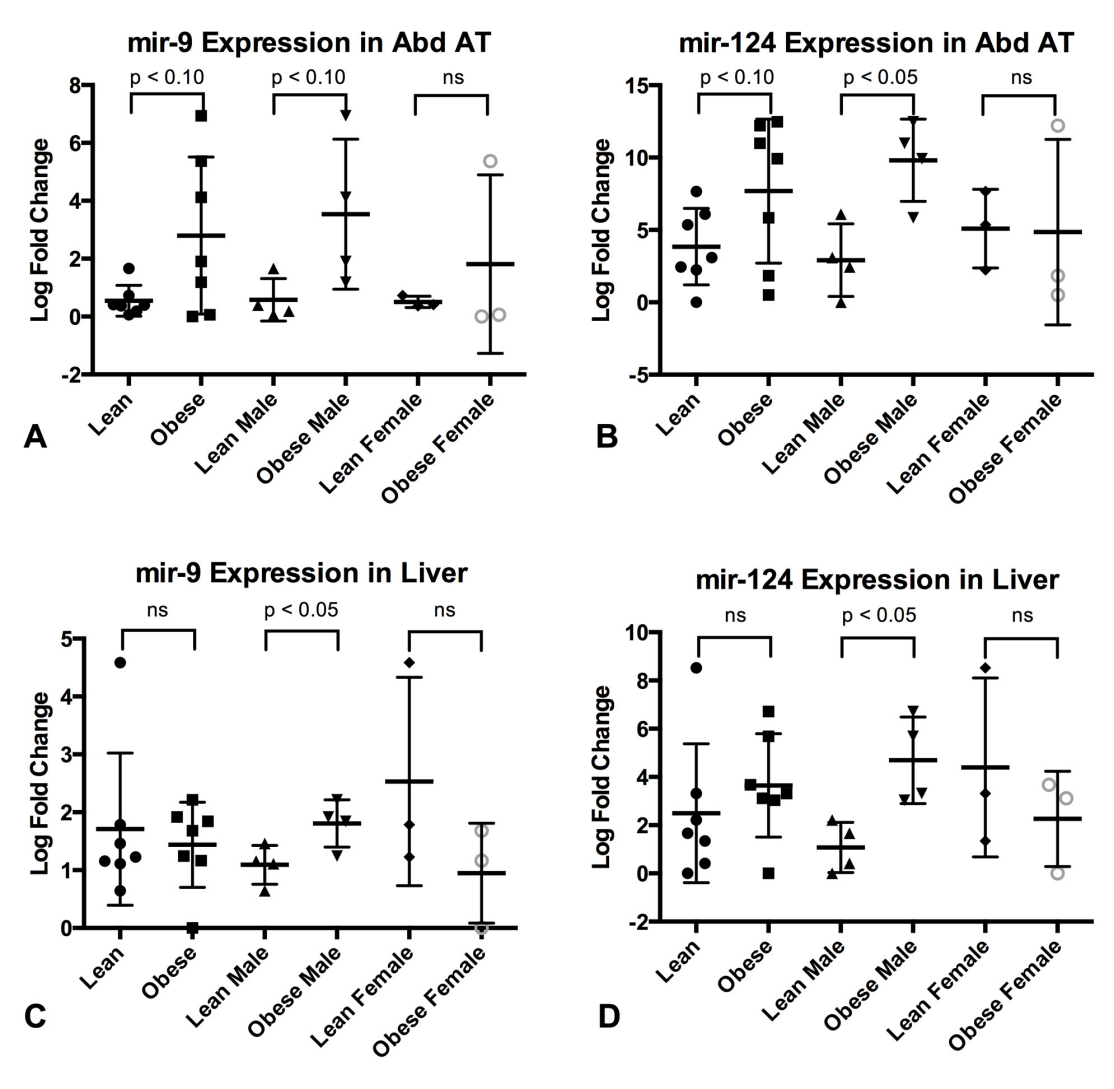
Expression of mir-9 and mir-124a in Abdominal Adipose Tissue and Liver. qPCR expression of mir-9 and mir-124a in Abdominal Adipose Tissue (Abd AT) (A,B) and Liver (C,D). Column scatter plot of the groups Lean and Obese, Lean Male and Obese Male and Lean Female and Obese Female is shown for each of the two microRNAs in all three tissues. P-values calculated by Student’s t-test.

To further study the expression of mir-9 and mir-124a, the two miRNAs with the largest fold changes between the lean and obese group in the subcutaneous adipose tissue, qPCR was also performed on cDNA from abdominal adipose tissue, liver and muscle from the same animals as in the study of subcutaneous adipose tissue. The data is presented as column scatter plots in Fig 3. Mir-9 and mir-124a were expressed in all three tissues.

The expression patterns differ, however, between the two genders. In abdominal fat the expression of both mir-9 and mir-124a was found to be more highly expressed in obese males (mir-124a FC: 119.8; p-value = 0.0108, mir-9 FC: 7.8; p-value = 0.07) and both were significantly more highly expressed in the liver of obese males (mir-124a FC: 12.3; p-value = 0.013, mir-9 FC: 1.6; p.value = 0.036) while no difference in expression could be detected in the females. In muscle no difference in expression was observed. All data is available in S6 Dataset.

Analysis of mir-9 and mir-124a target genes has not previously been performed in adipose tissue. In the literature, many target genes for both miRNAs have been verified with methods such as western blot, qPCR and luciferase transfer assay, and as described in materials and methods all verified targets from miRTarBase are listed in S7 Dataset. A selection, based on tissue expression and obesity relevance, of the target genes from S7 Dataset, was analyzed using qPCR. None of the selected targets were significantly down regulated in the obese group. Results from analysis of the data in males and females separately yielded a similar pattern (see S8 Dataset).

## Discussion

The sample population in this study consists of 14 animals of which 8 were male and 6 were females. It can be seen from the anthropomorphic measurements in Table 1, that the female pigs appear to be more obese than the male pigs. This has been observed in another study where female minipigs gain weight faster and get more obese than male minipigs. Both will however, when fed unrestricted, become obese [48]. Since Göttingen minipigs are a part of the genetic background for the pig model used in this study, this finding is not surprising. The male pigs have significant differences in their ASAT/ALAT ratio, a measure of liver damage [49]. Obesity is associated with nonalcoholic fatty liver disease (NAFLD), a condition where the liver store excess amount of fat and thus contribute further to the complications of obesity [50]. In human studies it has been observed that males have a higher tendency to NAFLD [51]. There is a significant difference in age between the lean female pigs and the obese female pigs, i.e. the obese female pigs are older than the lean female pigs. Thus, it cannot be ruled out that the obesity of the female pigs and the differential expression of miRNA are due to maturity of the pigs. However, in the male pigs the picture is reversed, i.e. the lean pigs are older than the obese pigs, however, this difference is non-significant.

In this study Illumina sequencing of the small RNA fraction was performed on RNA from subcutaneous adipose tissue of all 14 pigs. The top 10 expression of miRNAs from all 14 pigs (Fig 1) is consistent with previous studies of subcutaneous adipose tissue expression studies in pigs [17,20] and humans [47].

Differential expression in the sequencing data from the lean and obese pigs was calculated using the DESeq2 package in R and revealed six significantly differentially expressed miRNAs between the two groups: mir-9-5p, mir-124a-3p, mir-9-3p, mir-199a-5p, mir-489-3p and mir-34c-3p. (Table 3). qPCR validation of these results in general confirms the expression pattern, however, statistical significant differential expression was only observed for two of the six miRNAs, mir-9 and mir-124a (Table 6, Fig 2). Both these miRNAs were up regulated in the obese state, both in the population as a whole and if divided by gender. Both mature arms of mir-9 were significantly differentially expressed in the sequencing data, but only the mir-9-5p was tested in qPCR.

This is, to our knowledge, the first study of subcutaneous adipose tissue of lean versus obese subjects where mir-9 and mir-124a have been shown to be significantly up regulated in the obese subjects with large fold changes compared to the lean subjects. However, both miRNAs have been observed in other tissues and in cell lines in relation to inflammation and obesity-derived diseases, with similar results pointing towards overexpression in high lipid or high inflammation states, which correlates well with our findings. An example is that mir-9 and mir-124 are slightly up regulated in the blood of diabetic patients compared to non-diabetic controls [52].

Obesity leads to increased inflammation involving macrophage infiltration of the adipose tissue [2]. In a study of macrophages in lung inflammation, mir-124 expression was up regulated in macrophages by IL4 and IL13 stimulation as well as by allergy induced inflammation [53]. In the present study, where all cells in the adipose tissue have been included for the extraction of RNA, it might be that the increased mir-124a expression in this tissue is due to the presence of macrophages. On the other hand, adipocytes have also been proven to express mir-124 [54].

Mir-124 expression has previously been linked to weight gain. In a study of mice blastocysts injected with mir-124, the injection resulted in a 30% weight increase of the mice pups compared to control mice pups. This effect lasted for generations. The suspected target gene was Sox9 and embryonic overexpression of mir-124 lead to overexpression of this target gene in the adult mouse, presumably to compensate for the abnormal embryonic miRNA profile [55]. Sox9 is a transcription factor, which is highly expressed in adipose tissue derived stem cells and its down regulation is essential for differentiation into adipocytes [56,57]. Mir-124 is also targeting ADIPOR2 –a receptor for the protein hormone adiponectin, which is secreted in adipose tissue [58]. ADIPOR2 expression is negatively correlated with obesity traits as it is speculated to have a protective role in insulin resistance, since expression increased when obese subjects undergo physical exercise [59].

Mir-9 expression has also been linked to the obesity related diseases diabetes and inflammation. Mir-9 over-expression in β-cells induces exocytosis and thereby insulin release by targeting the transcription factor Onecut, which negatively regulates granuphilin. Granuphilin is located on β-cell secretory vesicles and is involved in the secretory response of the insulin-producing cells [60]. Mir-9 expression is also induced in monocytes and neutrophils upon activation of the immune receptors TLR4, TLR2 and TLR7/8 and by the pro-inflammatory cytokines TNF-a and IL-1B. NFKB1, an inflammation relevant transcription factor, is identified as the potential target gene [61].

The overexpression of mir-9 and mir-124 in the adipose tissue of obese pigs may also contribute to the lipid accumulation in the adipocytes. As described in humans, obese pigs have larger adipocytes, due to lipid accumulation, than lean pigs [62].

Hepatic stellate cells (HSCs) are specialized liver cells that, like adipocytes, accumulate lipid droplets, which they lose upon activation caused by liver damage. Inactivated lipid carrying HSCs have higher mir-9 and mir-124 expression than activated HSCs that carry no lipids [63].

The extraordinary high fold changes of mir-9 and mir-124a in the obese compared to the lean subjects prompted us to perform qPCR in three additional obesity relevant tissues: abdominal adipose tissue, liver and skeletal muscle (see Fig 3). While no differential expression was observed in skeletal muscle, gender specific differential expression was observed in both abdominal adipose tissue and liver. In abdominal adipose tissue both mir-9 and mir-124a was expressed at a higher level in the obese male pigs, (p-value = 0.07 and 0.01 respectively). Mir-9 and mir-124a were both significantly differentially expressed in the liver of obese males, while there was no significant difference in the females. The gender differences in expression observed in abdominal adipose tissue and liver might be related to the differences in fat storage observed between males and females [8].

A selection of target genes for mir-9 and mir-124 (list in S7 Dataset) were tested by qPCR but none of them were significantly suppressed in the obese group (S8 Dataset). This illustrates the difficulty in assessing miRNA:Target interactions experimentally on the RNA level. The changes in mRNA level upon miRNA binding may be very subtle or even non-existent as the changes are only measurable at the protein level, due to the mRNA being transcribed in normal levels, and the miRNA regulation in mammals is often limited to suppression of translation [23].

Of the other six differentially expressed miRNAs in the sequencing experiment, mir-199a-5p, which was significantly down regulated in obese subjects in the sequencing data, was also down regulated when assessed by qPCR, but did not reach significance. Mir-199a-5p has been identified as differentially expressed in subcutaneous adipose tissue from humans, where it was up regulated in obesity [64]. The other mature arm of mir-199a, mir-199a-3p, has been identified as down regulated upon weight loss in morbidly obese human patients [65].

Two of the additional differentially expressed miRNAs from the sequencing data, mir-34c and mir-489 could not be analyzed by qPCR due to very low expression in the majority of the samples. In other studies mir-34c has been shown to be expressed during adipogenesis of 3T3-L1 adipocytes and in murine brown adipocytes. It has, however, not been found differentially expressed in subcutaneous adipose tissue in humans [66,67]. Mir-489 is expressed in the adipocyte precursor mesenchymal stem cells, where its expression inhibits differentiation into osteoblasts [68]. It has also been linked to maintaining cell quiescence in muscle stem cells [69].

Apart from the miRNAs differentially expressed in the sequencing study, a couple of other miRNAs were also assessed in subcutaneous adipose tissue by qPCR. Mir-99a was significantly down regulated in obese female pigs in the sequencing study, but it is significantly up regulated in obese females and significantly down regulated in obese males in the qPCR study. The up regulation of mir-99a in qPCR of the obese female pigs is consistent with a study in subcutaneous adipose tissue of obese women where mir-99a also was significantly up regulated in the obese state [64]. Mir-99a is also up regulated during adipocyte differentiation into mature adipocytes [64,67]. Interestingly, in the sequencing results mir-99a was significantly down regulated in obese females. This result, together with the results on mir-10b are examples of inconsistent results between NGS and qPCR as has also reported by others [19].

Mir-10b is, apart from being the most expressed miRNA with more than 32% of the total read count in the sequencing study (Fig 1), also differentially expressed in the obese female pigs (Tables 5 and 6) where it is down regulated in female obesity according to the sequencing study and up regulated in the qPCR study. However, the fold changes are small, and this small differential expression may only have minor biological significance. Mir-10b is up regulated in adipocyte differentiation [67], which together with the high expression in adipose tissue suggests a housekeeping regulatory function in this tissue. Mir-103 is up regulated in the obese female pigs in both the sequencing study and the qPCR study (Tables 5 and 6). With a fold change of 1.73 the differential expression might have a moderate impact on protein expression in adipose tissue of the female pigs. Mir-103 has previously been found to be expressed during adipocyte differentiation and to accelerate adipogenesis [70,71].

In conclusion, sequencing the small RNA fraction of subcutaneous adipose tissue reveals differences in miRNA expression between lean and obese pigs with some gender specific differences. QPCR studies confirm some of these differences, in particular for mir-9 and mir-124a which are significantly differentially expressed with large fold changes. Mir-9 and mir-124a are significantly up regulated in subcutaneous adipose tissue of obese pigs, independently of gender. Additionally, only mir-124 is significantly up regulated in abdominal adipose tissue of male obese pigs and both mir-9 and mir-124a are up regulated in the liver of obese male pigs. These results indicate that gender differences in fat storage are potentially regulated by miRNAs.

## Supporting Information

S1 Dataset MirDeep2 Output of Counts from all 14 Animals. (XLSX)

S2 Dataset DESeq2 Analysis Lean vs Obese All Animals. (XLSX)

S3 Dataset DESeq2 Analysis Lean vs Obese Male Animals. (XLSX)

S4 Dataset DESeq2 Analysis Lean vs Obese Female Animals. (XLSX)

S5 Dataset Student’s t-test of miRNA qPCR Expression in Subcutaneous Adipose Tissue. (XLSX)

S6 Dataset Student’s t-test of mir-9 and mir-124a qPCR Expression in Liver and Abdominal Adipose Tissue. (XLSX)

S7 Dataset MiRTarbase Verified mir-9 and mir-124 Target Genes. (DOCX)

S8 Dataset Student’s t-test of target mRNA qPCR Expression in Subcutaneous Adipose Tissue. (XLSX)

S9 Dataset Boxplots for the raw Ct expression of reference genes in obese and lean animals. (XLSX)
