# Supplementary material for: Gender and Obesity Specific MicroRNA Expression in Adipose Tissue from Lean and Obese Pigs
Source: PLoS One. 2015 Jul 29;10(7):e0131650. doi: 10.1371/journal.pone.0131650 (PMC4519260; doi:10.1371/journal.pone.0131650)
Supplement: S7 Dataset — (DOCX) [file pone.0131650.s007.docx]

**S7 Dataset. Verified target list from miRTarBase.**

Lists of targets verified in human studies by luciferase reporter assay, qPCR or western blot from miRTarbase (Targets verified by strong evidence was downloaded at <http://mirtarbase.mbc.nctu.edu.tw/php/download.php>).

**Mir-9 target list**

RCOR1

ITGB1

MMP13

REST

CDH1

POU2F2

BCL6

ETS1

RAB34

BACE1

PRDM1

FOXO1

NFKB1

NTRK3

NR2E1

ONECUT2

CDX2

SIRT1

TGFBI

SOCS5

ID2

FOXO3

CCND1

VIM

CHMP2B

STMN1

GRN

CD34

**Mir-124 target list**

EFNB1

NR3C2

BACE1

ADIPOR2

MTPN

CEBPA

IL6R

ROCK2

RELA

CDK4

CDK6

AHR

SLC16A1

IQGAP1

SNAI2

LAMC1

CTDSP1

ITGB1

HMGA1

AR

RDH10

ELK3

CDK2

CCL2

PEA15

EZH2

NR3C1

VIM

SMYD3

E2F6

NFKBIZ

FXN

MECP2
